# Epidural analgesia and postoperative complications in colorectal cancer surgery. An observational registry‐based study

**DOI:** 10.1111/aas.14101

**Published:** 2022-06-27

**Authors:** Rune P. Hasselager, Jesper Hallas, Ismail Gögenur

**Affiliations:** ^1^ Center for Surgical Science Zealand University Hospital Koege Denmark; ^2^ Clinical Pharmacology and Pharmacy Odense University Hospital Odense Denmark; ^3^ Department of Clinical Medicine University of Copenhagen Copenhagen Denmark

## Abstract

**Background:**

In colorectal cancer, surgical resection is fundamental for curative treatment. Epidural analgesia mitigates the perioperative physiologic stress response caused by surgery, and reduction in perioperative stress may reduce postoperative complications. Nevertheless, epidural analgesia also causes hypotension and lower limb motor weakness that can impair postoperative recovery. Here, we aimed to assess the association between epidural analgesia and postoperative complications after colorectal cancer surgery.

**Methods:**

We identified patients undergoing colorectal cancer surgery 2008–2018 in Denmark in the Danish Colorectal Cancer Group Database and obtained anaesthesia data from the Danish Anaesthesia Database. The Danish National Prescription Registry was used to obtain data on prescriptions filled preoperatively reflecting current comorbidities. Databases were linked using the Danish Central Person Registry number and the operation day. Patients were classified according to preoperative insertion of an epidural catheter for analgesia. Confounders were adjusted by propensity score matching. Logistic regression was used to compute effect estimates of epidural analgesia on postoperative complications.

**Results:**

We identified 19 932 individuals undergoing colorectal cancer surgery with available anaesthesia data. Propensity score matching yielded 5691 individuals in each group with balanced preoperative covariates. In the epidural analgesia group 1400 (24.6%) experienced complications compared with 1453 (25.5%) without epidural analgesia. We found no statistically significant association between epidural use and postoperative complications (OR 0.95, 95% CI 0.87–1.04).

**Conclusion:**

In total, in this observational study based on Danish registries, we found no association between epidural analgesia and postoperative complications after colorectal cancer surgery.

**Editorial Comment**

The findings reported here are based on a substantial information collected during 10 years from three registers, the Danish Colorectal Cancer Group Database, the Danish Anaesthesia Database and the Danish National Prescription Registry. The results do not show an association between perioperative epidural use and benefit defined as complication risk in 2‐day fast‐track pre‐, peri‐ and postoperative care. This was uncontrolled case material, and so there is some risk for bias or confounding factors affecting results.

## 1 INTRODUCTION

Surgical resection is the main curative treatment for colorectal cancer.^1^ Each year, millions of surgical tumour resections are performed worldwide, and the global demand for cancer surgery is increasing.^2^ Yet, postoperative complications are frequent and often lead to poor recovery.^3^ With an ageing and increasingly frail population undergoing cancer surgery, the perioperative period is increasingly complex, and cancer surgery is becoming a significant global health burden.^4^ Therefore, there is a need for knowledge on interventions that can enhance the postoperative course for patients undergoing colorectal cancer surgery.

Perioperative stress denotes the physiologic response to surgery across inflammatory, immunologic and organ‐specific systems. Primarily, the perioperative stress response is activated by afferent nerve impulses from the surgical wound.^5^ In the brain, these impulses activate the hypothalamo‐pituitary–adrenal axis, which induces a sympathetic neuroendocrine response dominated by cortisol and catecholamines. The excretion of hormones by the hypothalamo‐pituitary–adrenal axis and direct activation mediated by damage associated molecular patterns from the surgical wound causes an inflammatory phase followed by a phase of immunosuppression that affects all organ systems.^6^ The perioperative stress response supports immediate wound healing by activation of coagulation and inflammatory cascades; however, extensive surgical stress affects physiological homoeostasis of multiple organ systems.^7^ Thus, the extent of perioperative stress may lead to postoperative impaired recovery and complications of both the surgical wound and other organ systems.^8^

Epidural analgesia, where local anaesthesia is used to block afferent nerve fibres in the epidural space, are effective in reducing pain after colorectal surgery.^9^ By blocking afferent nerve fibres from the surgical wound to the brain, epidural analgesia also reduces the activation of the hypothalamo‐pituitary–adrenal axis, and thereby, reduces the perioperative physiologic stress.^10^ Moreover, efferent activation of the sympathetic nerve system from the hypothalamus is blocked by epidural analgesia.^10^ It has been proposed that epidural analgesia reduces complications through these mechanisms; however, epidural analgesia has side effects that are noteworthy to consider. Due to sympatholytic vasodilation, epidural analgesia often causes hypotension and blocked nerve transmission can result in lower limb motor weakness and bladder dysfunction, and epidural analgesia may thereby impair early mobilization after surgery leading to complications.^11^ As there are features of epidural analgesia pulling in opposite directions in terms of postoperative recovery, there is a need for detailed insights into the beneficial or detrimental effects of epidural analgesia after colorectal cancer surgery.

We sought to answer the question: is there any benefit of epidural analgesia on postoperative complications after colorectal cancer surgery? We hypothesized that in colorectal cancer surgery, epidural analgesia was related to reduced rates of postoperative complications mediated by a reduced physiologic stress response to surgery. Thus, we sought to estimate if epidural analgesia was associated with postoperative complications by leveraging Danish databases of routinely and prospectively collected data in patients undergoing colorectal cancer surgery. Moreover, we aimed to explore associations between epidural analgesia and specific postoperative complications.

## 2 METHODS

This was an observational study of patients undergoing colorectal cancer surgery 2008–2018 based on routinely and prospectively collected data from Danish registries. Since the study was based on registry data, no informed consent from participants or approvals from ethical review boards were required according to Danish law. Data processing was approved by the Danish Health Data Authority and the Danish Data Protection Agency (file no. 2012‐58‐0003, REG‐038‐2017) and the study was reported according to STROBE and RECORD guidelines.^12^, ^13^ A checklist of the guideline items is available as Table S1.

### 2.1 Data Sources

The cohort was identified using the Danish Colorectal Cancer Database,^14^ which contains data on all patients operated for colorectal cancer in Denmark. Data on the type of anaesthesia used were obtained from the Danish Anaesthesia Database.^15^ Besides data on the type of anaesthesia and surgery, these databases contain detailed information on patient characteristics, intraoperative events and postoperative recovery entered by the clinicians responsible for patient treatment. Since the quality of registrations of epidural analgesia was poor during first years after the Danish Anaesthesia Database was established in 2004, we chose to include patients from 2008 onwards only.

To describe the population in further detail, we included patient level data of reimbursed prescriptions from the Danish National Prescription Registry,^16^ which contain data of prescriptions from all pharmacies in Denmark. Data on postoperative mortality were obtained from the Danish Civil Registration System.^17^ Data sources were linked using the unique Central Person Registration number that is assigned to all Danish residents.

### 2.2 Setting and participants

We included patients undergoing colorectal cancer surgery under general anaesthesia in Denmark 2008–2018. We excluded endoscopic local resections and operations where no tumour resection was performed. During the study period, all colorectal cancer surgery in Denmark was performed in public hospitals reporting to the Danish Colorectal Cancer Group Database. During the study period, the use of minimally invasive surgery increased from about 25%–80% and the median length of postoperative hospital stay decreased from 7 to 4 days (Figures S1 and S2). During the study period, the level of the epidural catheters was categorized as thoracic for 80% of patients undergoing colorectal cancer surgery.

All included patients received general anaesthesia for surgery and were classified according to preoperative insertion of an epidural catheter. Thus, patients receiving preoperative epidural analgesia registered in the Danish Anaesthesia Database constituted the treatment group, and the control group were patients not receiving epidural analgesia preoperatively. Spinal anaesthesia in combination with general anaesthesia is rarely used in Denmark and the duration and physiological impact is substantially different from epidural analgesia. Therefore, we excluded patients undergoing spinal anaesthesia without epidural analgesia.

### 2.3 Outcomes

The primary outcome was any postoperative complications within the first 30 days after colorectal cancer surgery. Since epidural analgesia may be protective against some complications and detrimental for others, we explored associations with specific postoperative complications. We included the following surgical complications: ‘any surgical complication’, complications related to wound healing (anastomotic leakage and wound dehiscence), infection (wound abscess and intraabdominal abscess) and other surgical complications (stoma complications requiring surgical treatment, ileus, haemorrhage and surgical complication not classified elsewhere). The following categories of medical complications were included: ‘any medical complication’, thromboembolic events (stroke, myocardial infarction, pulmonary embolism, deep vein thrombosis and arterial embolism), infections (pneumonia and sepsis), organ failure (respiratory failure, heart failure and kidney failure) and other medical complications (aspiration to lungs and medical complications not classified elsewhere). Additionally, death within 30 days after surgery was included.

Based on relevant suggestions from peer‐reviewers, we chose to report postoperative length of postoperative hospitalization as an outcome. This was based on admission and discharge registrations from the Danish National Patient Registry,^18^ where we had access to all records with a cancer diagnose registered. Prolonged postoperative hospital stay indicates a postoperative course with postoperative complications or impaired recovery. Prolonged hospitalization was defined as being longer than 10 postoperative days.

Outcome definitions are described in detail in Table S2.

### 2.4 Other variables

Baseline covariates describing the population prior to surgery included age, sex, body mass index and lifestyle factors. Patient frailty is a phenotype that is often seen in aged or multi‐morbid patients characterized by reduced physiologic tolerance to stress.^19^ Since we expected frailty to be an important potential confounder, we included the following variables to reflect various aspects of the phenotype: disease history, assessed by the Charlson Comorbidity Index^20^; physical performance status, by the American Society of Anesthesiologists (ASA) Physical Status^21^; and current diseases requiring medication, assessed by recently filled prescriptions from the Danish National Prescription Registry. A comprehensive table of included variables and definitions are appended in Table S2.

### 2.5 Confounder adjustment

Propensity score matching was applied to account for confounding.^22^ Based on baseline characteristics, propensity scores were determined using logistic regression with epidural analgesia as outcome and all baseline covariates as exposure variables. To avoid including covariates that may lie in the causal pathway between exposure and outcome, we only included covariates that occurred before exposure to treatment.^23^ As the decision to install a preoperative epidural catheter is made before initiation of surgery, we did not include any intra‐ or postoperative covariates. The covariates are listed in Table 1 and include: age, sex, body mass index, tobacco use, alcohol consumption, Charlson Comorbidity Index, ASA Physical Status, prescriptions 3 months before surgery, preoperative cancer stage, tumour localization, neoadjuvant oncologic therapy, surgical approach, urgency, general anaesthesia type and year. The R package MatchIt^24^ was used to perform nearest neighbour matching with random order and a calliper of 0.75. Covariate balance after propensity score adjustment were evaluated using standardized mean differences.^25^ To ensure that covariates were sufficiently balanced, standardized mean difference was required to be below 0.1 for all covariates after matching.

**TABLE 1:** Characteristics of patients undergoing colorectal cancer surgery 2004–2018 included in study before and after propensity score matching

	Before PS matching			After PS matching		
	Epidural analgesia	No epidural analgesia	SMD	Epidural analgesia	No epidural analgesia	SMD
*N*	6339	13,593		5691	5691	
Demographics						
Age (IQR)	70 (63–77)	71 (63–77)	0.031	71 (63–78)	71 (63–77)	0.008
Male	3434 (54.2)	7291 (53.6)	0.011	3063 (53.8)	3090 (54.3)	0.010
BMI						
<18.5	249 (3.9)	411 (3.0)	0.127	215 (3.8)	223 (3.9)	0.018
18.5–25	2917 (46.0)	5537 (40.7)		2570 (45.2)	2598 (45.7)	
25–30	2138 (33.7)	5091 (37.5)		1950 (34.3)	1905 (33.5)	
>30	1015 (16.0)	2502 (18.4)		937 (16.5)	947 (16.6)	
Missing	20 (0.3)	52 (0.4)		19 (0.3)	18 (0.3)	
Charlson Comorbidity Index						
0	3871 (61.1)	8179 (60.2)	0.018	1507 (26.5)	1463 (25.7)	0.019
1	1121 (17.7)	2452 (18.0)		3655 (64.2)	3700 (65.0)	
2	688 (10.9)	1511 (11.1)		348 (6.1)	350 (6.2)	
>2	659 (10.4)	1451 (10.7)		181 (3.2)	178 (3.1)	
ASA Physical Status						
I	1003 (15.8)	2124 (15.6)	0.008	907 (15.9)	893 (15.7)	0.010
II	3722 (58.7)	7987 (58.8)		3312 (58.2)	3335 (58.6)	
III	1502 (23.7)	3238 (23.8)		1366 (24.0)	1356 (23.8)	
IV	101 (1.6)	223 (1.6)		95 (1.7)	95 (1.7)	
Missing	11 (0.2)	21 (0.2)		11 (0.2)	12 (0.2)	
Lifestyle						
Tobacco						
Smoker	1288 (20.3)	2484 (18.3)	0.075	1113 (19.6)	1106 (19.4)	0.007
Non‐smoker	4854 (76.6)	10,797 (79.4)		4410 (77.5)	4411 (77.5)	
Missing	197 (3.1)	312 (2.3)		168 (3.0)	174 (3.1)	
Alcohol consumption (weekly units)						
0	1686 (26.6)	3322 (24.4)	0.092	1507 (26.5)	1463 (25.7)	0.018
1–21	4035 (63.7)	9196 (67.7)		3655 (64.2)	3700 (65.0)	
>21	395 (6.2)	716 (5.3)		348 (6.1)	350 (6.2)	
Missing	223 (3.5)	359 (2.6)		181 (3.2)	178 (3.1)	
Prescriptions filled 3 months preoperatively (ATC‐codes)						
Proton pump inhibitors (A02BC)	1227 (19.4)	2494 (18.3)	0.026	1089 (19.1)	1100 (19.3)	0.005
Antidiabetics (A10)	617 (9.7)	1338 (9.8)	0.004	553 (9.7)	561 (9.9)	0.005
Acetyl Salicylic Acid (B01AC06)	924 (14.6)	1830 (13.5)	0.032	822 (14.4)	830 (14.6)	0.004
Other Platelet Inhibitors (B01AC	301 (4.7)	767 (5.6)	0.040	282 (5.0)	287 (5.0)	0.004
Anticoagulants (B01A)	313 (4.9)	957 (7.0)	0.089	298 (5.2)	308 (5.4)	0.008
Digoxin (C01AA05)	165 (2.6)	318 (2.3)	0.017	137 (2.4)	132 (2.3)	0.006
Thiazides (C03)	1092 (17.2)	2295 (16.9)	0.009	987 (17.3)	981 (17.2)	0.003
Beta blockers (C07)	940 (14.8)	2133 (15.7)	0.024	865 (15.2)	862 (15.1)	0.001
Calcium Channel Blockers (C08)	911 (14.4)	2106 (15.5)	0.031	837 (14.7)	858 (15.1)	0.010
Drugs acting on renin‐angiotensin system (C09)	1650 (26.0)	3723 (27.4)	0.031	1503 (26.4)	1516 (26.6)	0.005
Lipid lowering drugs (C10)	1381 (21.8)	3209 (23.6)	0.044	1264 (22.2)	1304 (22.9)	0.017
Oestrogen hormone replacement (G03C)	255 (4.0)	582 (4.3)	0.013	235 (4.1)	224 (3.9)	0.010
Corticosteroids for systemic use (H02)	204 (3.2)	403 (3.0)	0.015	175 (3.1)	170 (3.0)	0.005
Non‐steroid anti‐inflammatory drugs (M01A)	455 (7.2)	977 (7.2)	<0.001	413 (7.3)	395 (6.9)	0.012
Urate lowering drugs (M04)	112 (1.8)	298 (2.2)	0.031	106 (1.9)	102 (1.8)	0.005
Bisphosphonates (M05BA, M05BB)	223 (3.5)	434 (3.2)	0.018	198 (3.5)	200 (3.5)	0.002
Opioids (N02A)	832 (13.1)	1449 (10.7)	0.076	714 (12.5)	721 (12.7)	0.004
Benzodiazepines (N05CD, N05CF)	602 (9.5)	1155 (8.5)	0.035	526 (9.2)	526 (9.2)	<0.001
Antidepressants (N06A)	475 (7.5)	975 (7.2)	0.012	421 (7.4)	418 (7.3)	0.002
Drugs for Obstructive Airway Diseases (R03)	586 (9.2)	1241 (9.1)	0.004	513 (9.0)	530 (9.3)	0.010
Number of different drugs dispensed 3 months preoperatively						
0–4	4167 (65.7)	9008 (66.3)	0.011	3737 (65.7)	3708 (65.2)	0.011
5–9	1795 (28.3)	3788 (27.9)		1617 (28.4)	1636 (28.7)	
≥10	377 (5.9)	797 (5.9)		337 (5.9)	347 (6.1)	
Preoperative UICC Stage						
IV	785 (12.4)	1146 (8.4)	0.131	667 (11.7)	670 (11.8)	0.003
I‐III	5461 (86.1)	12,268 (90.3)		4941 (86.8)	4936 (86.7)	
Missing	93 (1.5)	179 (1.3)		83 (1.5)	85 (1.5)	
Localization						
Colon	4100 (64.7)	9681 (71.2)	0.141	3813 (67.0)	3839 (67.5)	0.010
Rectum	2239 (35.3)	3912 (28.8)		1878 (33.0)	1852 (32.5)	
Preoperative oncologic treatment	878 (13.9)	1309 (9.6)	0.131	728 (12.8)	705 (12.4)	0.012
Intended surgical approach						
Open	3097 (48.9)	2766 (20.3)	0.628	2449 (43.0)	2376 (41.8)	0.026
Minimally invasive	3242 (51.1)	10,827 (79.7)		3242 (57.0)	3315 (58.2)	
Urgency						
Elective	5720 (90.2)	12,585 (92.6)	0.089	5090 (89.4)	5013 (88.1)	0.046
Acute	618 (9.7)	999 (7.3)		600 (10.5)	675 (11.9)	
Missing	<5 (0.0)	9 (0.1)		<5 (0.0)	<5 (0.1)	
Type of general anaesthesia						
Inhalation	2635 (41.6)	6508 (47.9)	0.166	2441 (42.9)	2467 (43.3)	0.017
Total intravenous anaesthesia	3661 (57.8)	6856 (50.4)		3207 (56.4)	3188 (56.0)	
Missing	43 (0.7)	229 (1.7)		43 (0.8)	36 (0.6)	
Year group						
2008–2011	2803 (44.2)	2902 (21.3)	0.583	2210 (38.8)	2061 (36.2)	0.068
2012–2015	2516 (39.7)	5806 (42.7)		2461 (43.2)	2653 (46.6)	
2016–2018	1020 (16.1)	4885 (35.9)		1020 (17.9)	977 (17.2)	

Abbreviations: ASA, American Society of Anesthesiologists; ATC, anatomical‐therapeutical‐chemical classification; BMI, body mass index; PS, propensity score; SMD, standardized mean difference; UICC, Union for International Cancer Control.

### 2.6 Statistical analyses

Based on existing literature we expected that there would be around 15% postoperative complications.^3^ With a power of 80%, a significance level of 0.05 and a minimum clinically relevant difference between groups of 20%, we would need a sample size of at least 2036 individuals in each group.

Study group characteristics were presented as medians with inter‐quartile rages (IQRs) for continuous variables and absolute numbers with percentages for categorical variables. Using logistic regression, we determined effect estimates expressed as odds ratios (ORs) with 95% confidence intervals (CIs). Differences between groups outside CI were considered statistically significant.

After assessing the pattern of missing data, we classified our missing data to be ‘missing at random’. We used the missing data indicator method to account for missing data. Thus, missing data were categorized as sublevels of the specific covariate. Thereby, the distribution of missing data was equal between study groups after propensity score matching.

Analyses were performed using R version 3.6.3.

### 2.7 Subgroup analyses

In the following pre‐specified subgroups, we assessed the association between epidural analgesia and any postoperative complication: operation urgency (acute and elective), age (≥70 years and < 70 years), ASA Physical Status (I‐II and III‐IV), surgical approach (laparotomy and minimally invasive) and tumour localization (colon and rectum). In acute surgery, the inflammatory and immunologic response to surgery is initiated before insertion of epidural analgesia. Therefore, the effect on the surgical stress response may be substantially different from elective surgery. Patients older than 70 years and patients with ASA Physical Status III–IV may have a certain degree of frailty and be more susceptible to adverse outcomes caused by surgery and opioid use. These groups may potentially have increased benefit of epidural analgesia. Open surgery causes a substantially higher degree of surgical stress than minimally invasive surgery does, and therefore it the effects of epidural analgesia may be modified by the surgical approach. Lastly, the aetiology and the approach to surgical resection differs between colon and rectal tumours. Therefore, it was relevant to assess if the localization of the tumour caused any modification of the effect estimates between epidural analgesia and postoperative complications.

We also included a post‐hoc analysis of patients with and without obstructive pulmonary disease. Patients with respiratory disease are susceptible to respiratory complications. Improved analgesia by epidural analgesia may prevent development of postoperative complications in this specific subgroup. The group was defined by filled prescriptions of medication for obstructive pulmonary disease within 3 months before surgery. We conducted separate propensity score matchings for each subgroup with the same specifications as in the main analysis.

### 2.8 Sensitivity analyses

We performed a number of additional pre‐specified analyses of the cohort. First, we assessed impact of confounder adjustment on effect estimates by performing the study analyses without any confounder adjustment. Large discrepancy between the crude and confounder adjusted effect estimates reflect substantial influence of confounders. Additionally, even though the techniques used for general anaesthesia typically are determined before induction of anaesthesia, the decision to convert total intravenous anaesthesia to inhalational anaesthesia can be made during the operation. In theory, effects of epidural analgesia, such as hypotension, could result in a switch from total intravenous anaesthesia to inhalational analgesia. Thereby, it can be argued that the agent used for anaesthesia may be a mediator instead of a potential confounder in the analyses. Thus, we chose to perform a sensitivity analysis where this covariate was removed.

We added a number of post‐hoc analyses based on suggestions during peer‐review. First, to identify if there were any associations with the individual surgical departments, we performed a sensitivity analysis adjusting for surgical departments. Departments where less than 5% of all procedure occurred were categorized at ‘other surgical departments’. Moreover, as propensity score matching results in a reduction of the population of treated patients, we also assessed the outcomes using multivariable logistic regression with all baseline characteristics as covariates. Lastly, to assess what the largest possible impact of missing data could be on our effect estimates, we generated the most extreme distributions of the missing data in each direction. First, all missing data were allocated to the best category for patients undergoing epidural analgesia and the worst possible for the control group. Second, we performed the analyses in the complementary scenario with missing data categorized into the worst category for the epidural analgesia group and the best for the control group. For the variables with missing data, the best versus worse categories were: body mass index (18.5–25.0 vs >30), ASA Physical Status (I vs IV),tobacco use (non‐smoker vs smoker), alcohol consumption (0 vs >21 weekly units), preoperative cancer stage (Union for International Cancer Control Stage I–III vs IV), operation urgency (elective vs acute) and type of general anaesthesia (inhalation vs. total intravenous anaesthesia).

## 3 RESULTS

**FIGURE 1:**
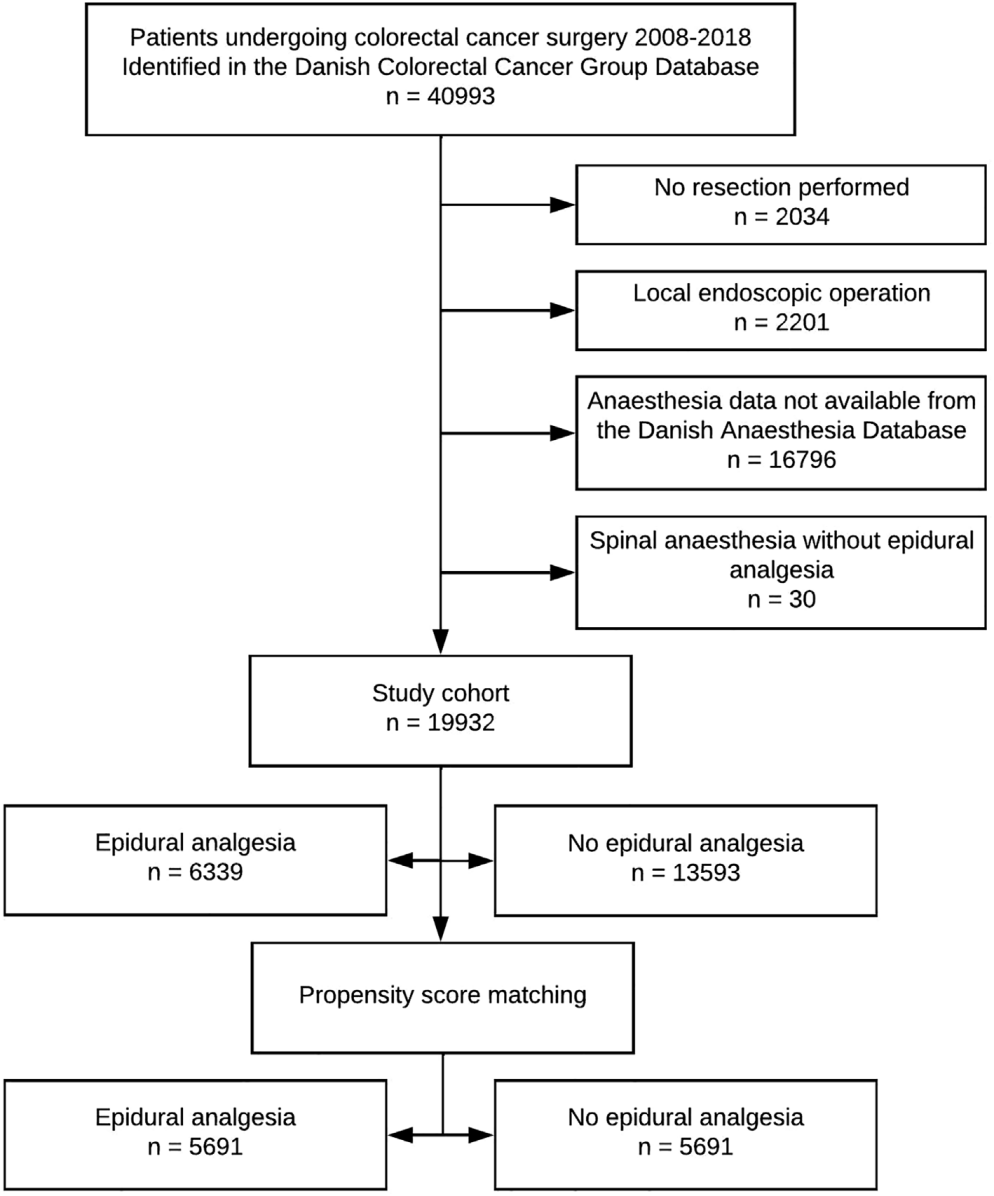
Flow diagram of study cohort

We identified 19,932 patients operated for colorectal cancer 2008–2018 with available data on the type of anaesthesia used. The study cohort is presented as a flowchart in Figure 1. The median age was 70 years (IQR 63–77 years) and 9207 (46.2%) were female. Of the total cohort, 6339 (31.8%) received epidural analgesia. We observed no missing data in the outcome variables except the post‐hoc analysis of length of hospital stay, where we were unable to obtain data for 753 (3.8%) individuals. There were low numbers of missing data in the variables ‘Tobacco use’, 509 (2.6%), and ‘Alcohol consumption’, 582 (2.6%). In the remaining covariates, the extent of missing data was negligible. More patients receiving epidural analgesia had lower body mass index, but higher frequency of Union of International Cancer Control Stage IV, rectal tumours, neoadjuvant oncologic treatment, intended open surgery and total intravenous anaesthesia compared with patients without epidural analgesia. The use of epidural analgesia decreased during the study period. In the period from 2008 to 2011 about half patients received epidural analgesia before surgery (range 48.2%–50.2%). This proportion fell to 15%–20% in the period from 2015 to 2018 (range 15.3%–20.0%) (Figure S3).

### 3.1 Propensity score matching and covariate balance

Propensity scores based on preoperative covariates were appropriately overlapping (Figure S4) and propensity score matching resulted in 5691 individuals in each group. As the highest standardized mean difference of all baseline covariates was 0.068, which was well below the threshold of 0.1, there was acceptable covariate balance. The covariate balance before and after matching is presented in Figure S5. The propensity score matched cohort resembled the total study cohort with a median age of 71 years (IQR 63–77 years) and 5229 (45.9%) females. Baseline characteristics before and after propensity score matching are presented in Table 1.

### 3.2 Postoperative complications

In the propensity score matched cohort, 2753 (25.1%) experienced at least one postoperative complication distributed as 1400 (24.6%) in the epidural analgesia group and 1453 (25.5%) in the group without epidural analgesia. This yielded an OR for postoperative complications of 0.95 (CI 0.87–1.04) for patients with epidural analgesia compared with the control group.

Surgical complications occurred in 1050 (18.5%) individuals with epidural analgesia and 1060 (18.6%) without epidural analgesia, OR 0.99 (CI 0.90–1.09). When specific surgical complications were assessed, we observed statistically significantly lower rates of postoperative haemorrhage, OR 0.46 (CI 0.33–0.63), and surgical complications not classified elsewhere, OR 0.82 (CI 0.67–0.99), in patients receiving epidural analgesia compared with general anaesthesia alone. We observed no statistically significant differences among the remaining surgical complications.

Medical complications occurred for 628 (11.0%) individuals in the epidural group and 654 (11.5%) in the control group with a corresponding OR of 0.96 (CI 0.85–1.07). Statistically significant differences were observed for arterial embolism, where less than five events were observed in the epidural analgesia group compared with seven events in the control group, OR 0.14 (CI 0.01–0.80), and kidney failure, OR 0.66 (CI 0.46–0.90).

After propensity score matching in patients receiving epidural analgesia, death occurred within the first 30 days postoperatively in 182 (3.2%) patients compared with 214 (3.8%) in the control group, OR 0.85 (CI 0.69–1.03). The length of postoperative hospital stay was 6 days (IQR 4–10 days) in the group with epidural analgesia compared with 6 days (IQR 3–10 days) in the group without epidural analgesia. We observed no difference in prolonged hospitalization (more than 10 days) between the groups.

**FIGURE 2:**
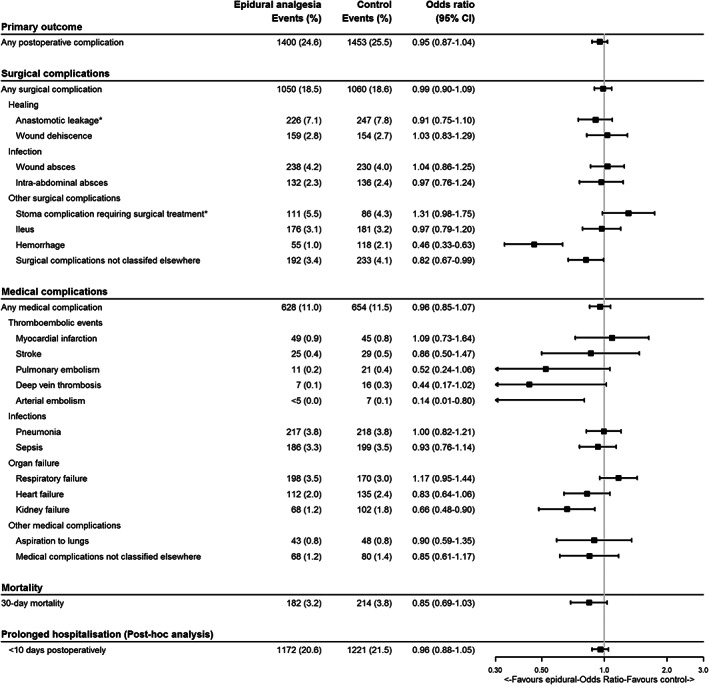
Forest plot of associations between epidural analgesia and postoperative complications after propensity score matching. Effect estimates were based on logistic regression. *The analyses of anastomotic leakage and stoma complications were based on populations with anastomosis or stoma performed. CI, confidence interval.

Results of analyses of postoperative complications are summarized in Figure 2.

### 3.3 Subgroup analyses

**FIGURE 3:**
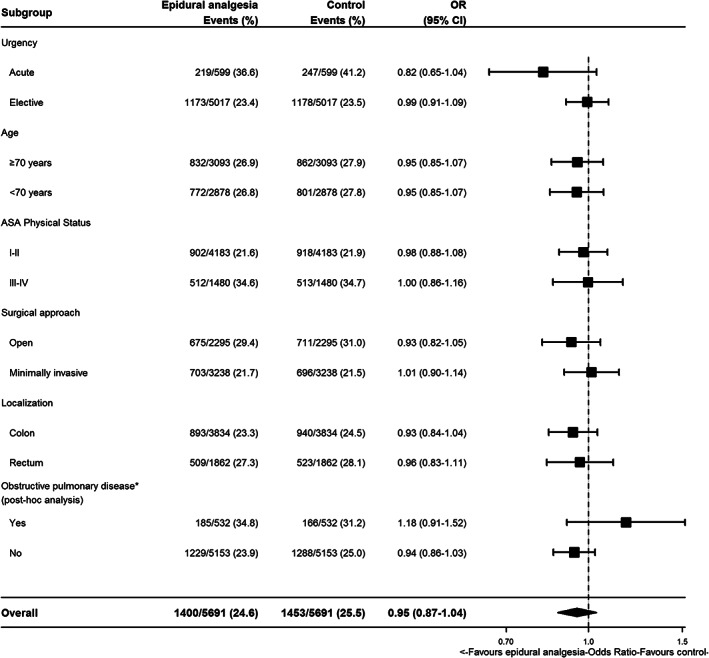
Forest plot of associations between epidural analgesia and postoperative complications in subgroups. The subgroups were bases on individual propensity score matchings. ASA = American Society of Anesthesiologists; CI, confidence interval; OR, odds ratio.

When the cohort was restricted according to the pre‐specified subgroups, we observed acceptable covariate balance after separate propensity score matchings in all subgroups. The effect estimates of epidural analgesia on postoperative complications were similar to the primary analysis for all subgroups. In the post‐hoc analysis of subgroups stratified according to obstructive pulmonary disease, we also found estimates with confidence intervals overlapping the estimate of the main analysis (Figure 3).

### 3.4 Sensitivity analyses

In the crude analysis without any confounder adjustment, we observed different effect estimates compared with the main analysis. In patients with epidural analgesia, there were higher rates of postoperative complications, OR 1.13 (CI 1.05–1.21), compared with the control group. Additionally, there were statistically significantly higher frequencies of both surgical and medical complications in the epidural analgesia group compared with control (Figure S6).

Multivariable regression including all baseline covariates resulted an OR of 0.95 (CI 0.88–1.03), which was similar to the main analysis. Similar to than main analysis epidural analgesia was related to lower risk of haemorrhage, surgical complications not classified elsewhere, and arterial embolisms. In contrast to the main analysis, we did not find a significant association between epidural analgesia and kidney failure (Figure S7). Exclusion of the type of general anaesthesia used during surgery also resulted in effect estimates that were comparable with the main analysis with OR 0.94 (CI 0.86–1.02). When we included hospitals into the propensity score model, the study groups were reduced by 1785 (31.4%) in each group and the OR was 0.92 (CI 0.81–1.00) for postoperative complications for the epidural analgesia group compared with control.

In the two scenarios where missing data were distributed skewed into the study groups, we assessed the highest possible impact of missing data on the effect estimates. Resulted in effect estimates that were similar to the main outcome (Figures S8 and S9).

## 4 DISCUSSION

In this observational study based on Danish registries of prospectively collected health data, we found no association between epidural analgesia and postoperative complications after colorectal cancer surgery. In explorative analyses of specific postoperative complications, we observed lower rates of postoperative haemorrhage and surgical complications not classified elsewhere related to preoperative epidural analgesia.

Other studies have focused on the effect of epidural analgesia on postoperative complications after abdominal surgery. In contrast to our results, a systematic review across various types of surgery under general anaesthesia found decreased risk of cardiovascular, respiratory and gastrointestinal complications related to epidural analgesia.^26^ Conversely, a systematic review focusing on effects of epidural analgesia in laparoscopic colectomy found no differences in postoperative surgical site infections, urinary tract infections, ileus or anastomotic leakage.^27^ Moreover, in keeping with our results, systematic reviews of abdominal surgery have found no difference in anastomotic leakage related to epidural analgesia.^28^, ^29^ The largest randomized trial investigating the effect of epidural analgesia on complications after major abdominal surgery found no effect on postoperative mortality or major complications but improved pain control the first 3 days after surgery.^30^ Additionally, small randomized trials of colorectal surgery have not demonstrated any difference in postoperative complications related to epidural analgesia after laparoscopic colorectal surgery.^31^, ^32^, ^33^

In our exploratory analyses of specific complications, we observed statistically significantly lower rates of postoperative haemorrhage. The reason for this finding is unclear but a possible explanation is that epidural analgesia causes hypotension, and thereby, prevents bleeding. Moreover, we observed lower rates of kidney failure in the epidural analgesia group than in the control group, which could be attributed to reduced surgical stress. Yet, this finding may likely be a chance finding since it was not observed in our sensitivity analysis using multivariable regression. Additionally, we observed reduced risk of arterial embolism related to epidural analgesia. The observed number of events for this outcome was very low, and this finding may also be a chance finding.

There are important limitations to keep in mind when interpreting the results of this study. First, the Danish Anaesthesia Database is only about 60% complete, and we did not succeed in obtaining anaesthesia data for the entire cohort undergoing colorectal cancer surgery during the study period. The quality of registrations of epidural analgesia and data completeness was poor in the initial years of the Danish Anaesthesia Database, which was initiated in 2004. Therefore, we restricted our analyses to the years 2008–2018. During this time period the use of epidural analgesia decreased, minimally invasive surgery became widespread and length of hospital stay decreased (Figures [Link], [Link]). This may represent implementation of Enhanced Recovery After Surgery programmes, which encourages the use of multimodal analgesia. Thus, epidural analgesia may largely have been replaced with multimodal analgesia during the study period. In our analysis, we adjusted for operation year and the intention to perform minimally invasive surgery. Still, Enhanced Recovery After Surgery programmes include multiple items that may be implemented to different degrees among departments, which we were unable to include in our analyses. Additionally, we did not have data on medications used and time of removal of epidural catheters. It is well known that some epidural catheters are misplaced and do not result in adequate analgesia. Therefore, our analyses should be considered as the association between postoperative complications and preoperative insertion of an epidural catheter with the intention of effective analgesia. Furthermore, we considered frailty to be a key confounder. However, there is no consensus on how to measure frailty and it is challenging to assess using registry data.^34^ We chose to include the combination of physical status, measured by ASA Physical Status, disease history, assessed by Charlson Comorbidity Index, and current medical conditions requiring medical treatment, reflected by recently filled prescriptions. There may be relevant factors related to frailty that are not included, and therefore, a risk of residual confounding. Lastly, even though all outcomes were entered into the Danish Colorectal Cancer Group Database by the surgeon responsible for surgery, there is a risk of registration error although this risk is considered minimal based on a recent validation study.^35^

Moving forward, the role of epidural analgesia in colorectal surgery should be considered. While we did not find a benefit of epidural analgesia regarding postoperative complications after colorectal cancer surgery, there are other benefits of epidural analgesia. Primarily, epidural analgesia reduces postoperative pain and opioid requirements.^9^ Other studies found longer hospital stay related to epidural analgesia.^36^ Yet, in our analyses, we did not observe an increased risk of prolonged hospitalization related to epidural analgesia. Thus, for patients at high risk of severe postoperative pain or adverse effects of opioids, epidural analgesia may still be appropriate. Other types of truncal regional analgesia could also be indicated.^37^

In total, in this large observational registry‐based study, epidural analgesia was not associated with postoperative complications after colorectal cancer surgery.

## Supporting information


Figure S1

Figure S2

Figure S3

Figure S4

Figure S5

Figure S6

Figure S7

Figure S8

Figure S9

Table S1

Table S2
