# Supplementary figures and images for: Epidural analgesia and postoperative complications in colorectal cancer surgery. An observational registry‐based study
Source: Acta Anaesthesiol Scand. 2022 Jun 27;66(7):869–79. doi: 10.1111/aas.14101 (PMC9543440; doi:10.1111/aas.14101)

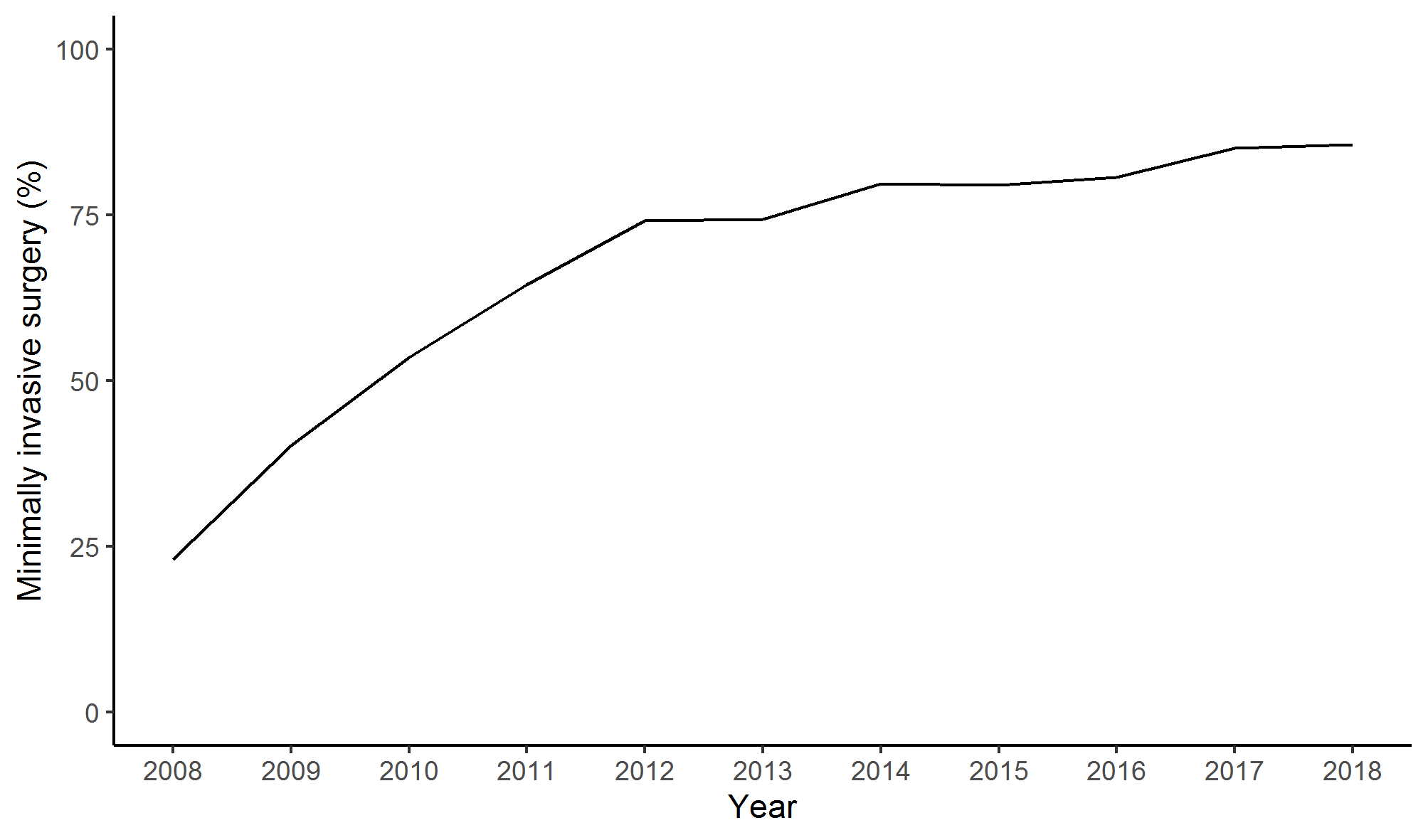

Supplement: Supplementary file 1 — Figure S1 [file AAS-66-869-s007.tif]

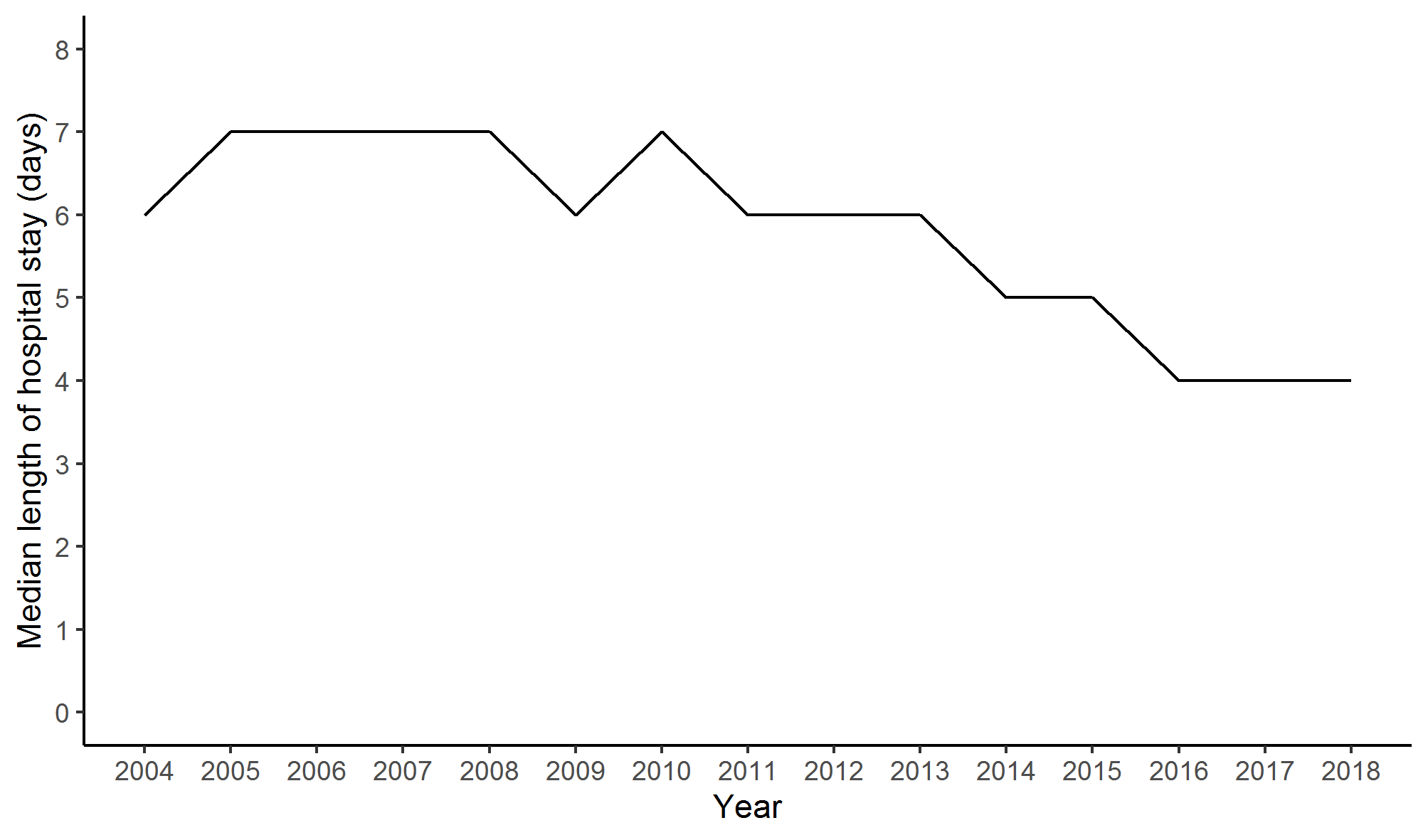

Supplement: Supplementary file 2 — Figure S2 [file AAS-66-869-s005.tif]

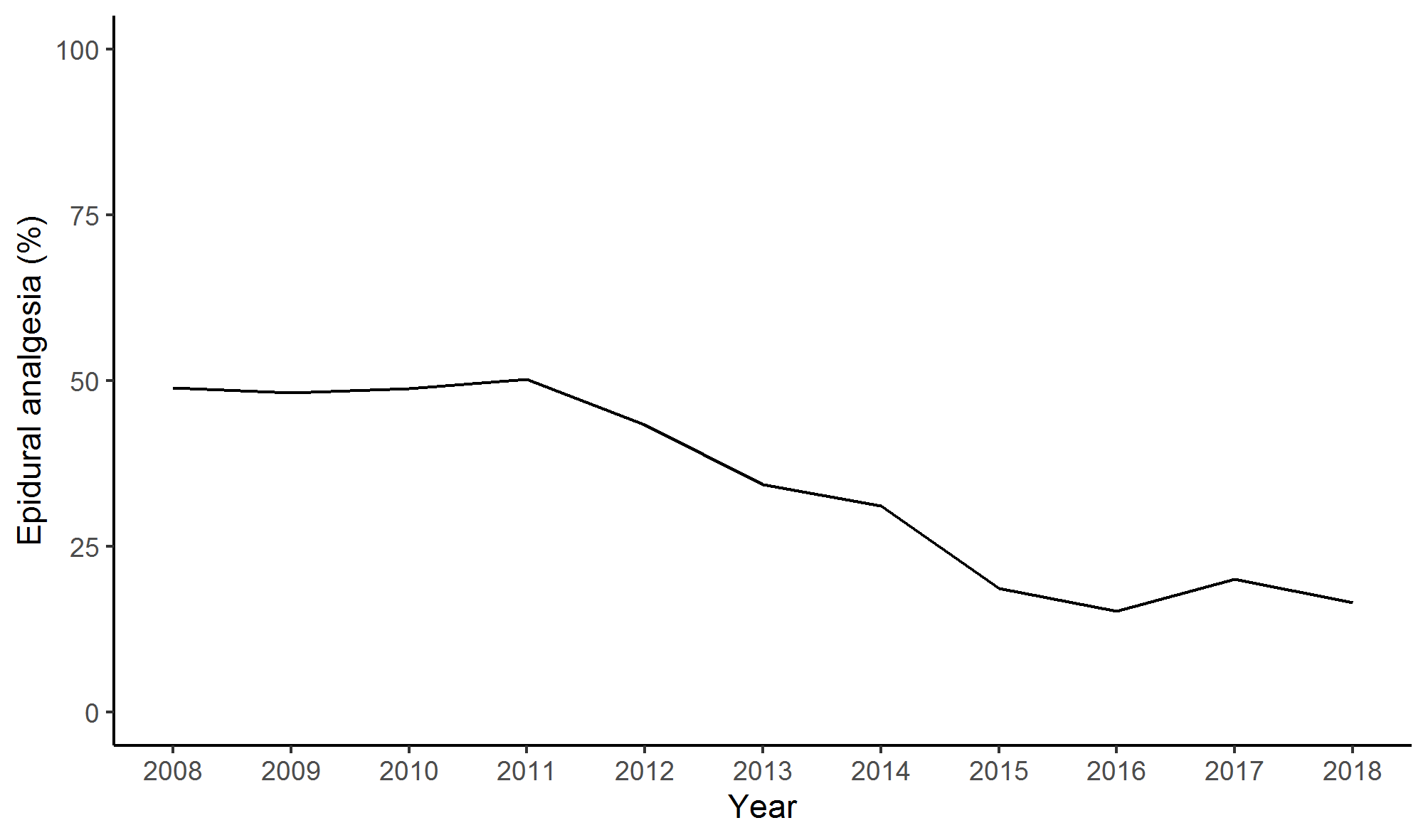

Supplement: Supplementary file 3 — Figure S3 [file AAS-66-869-s003.tif]

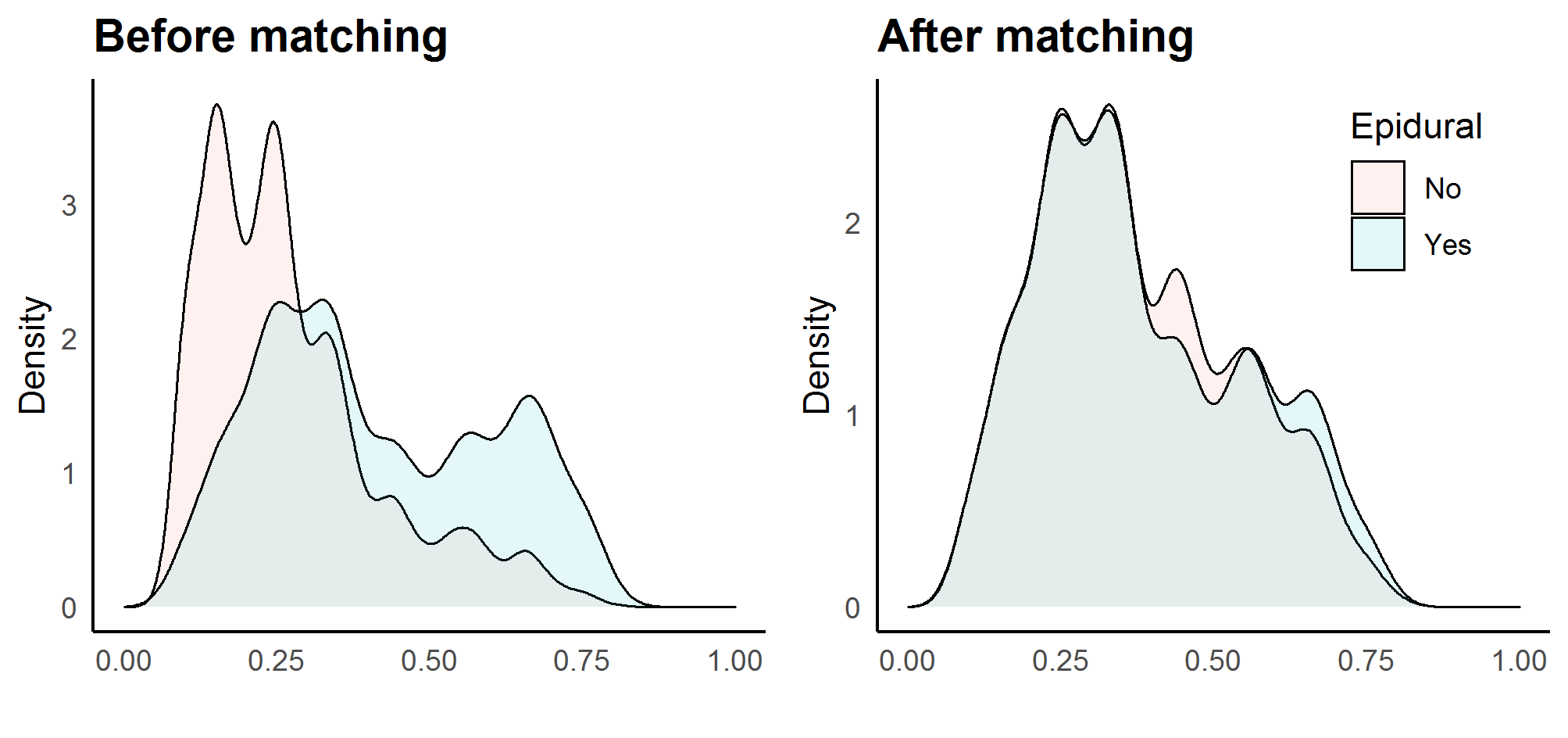

Supplement: Supplementary file 4 — Figure S4 [file AAS-66-869-s011.tif]

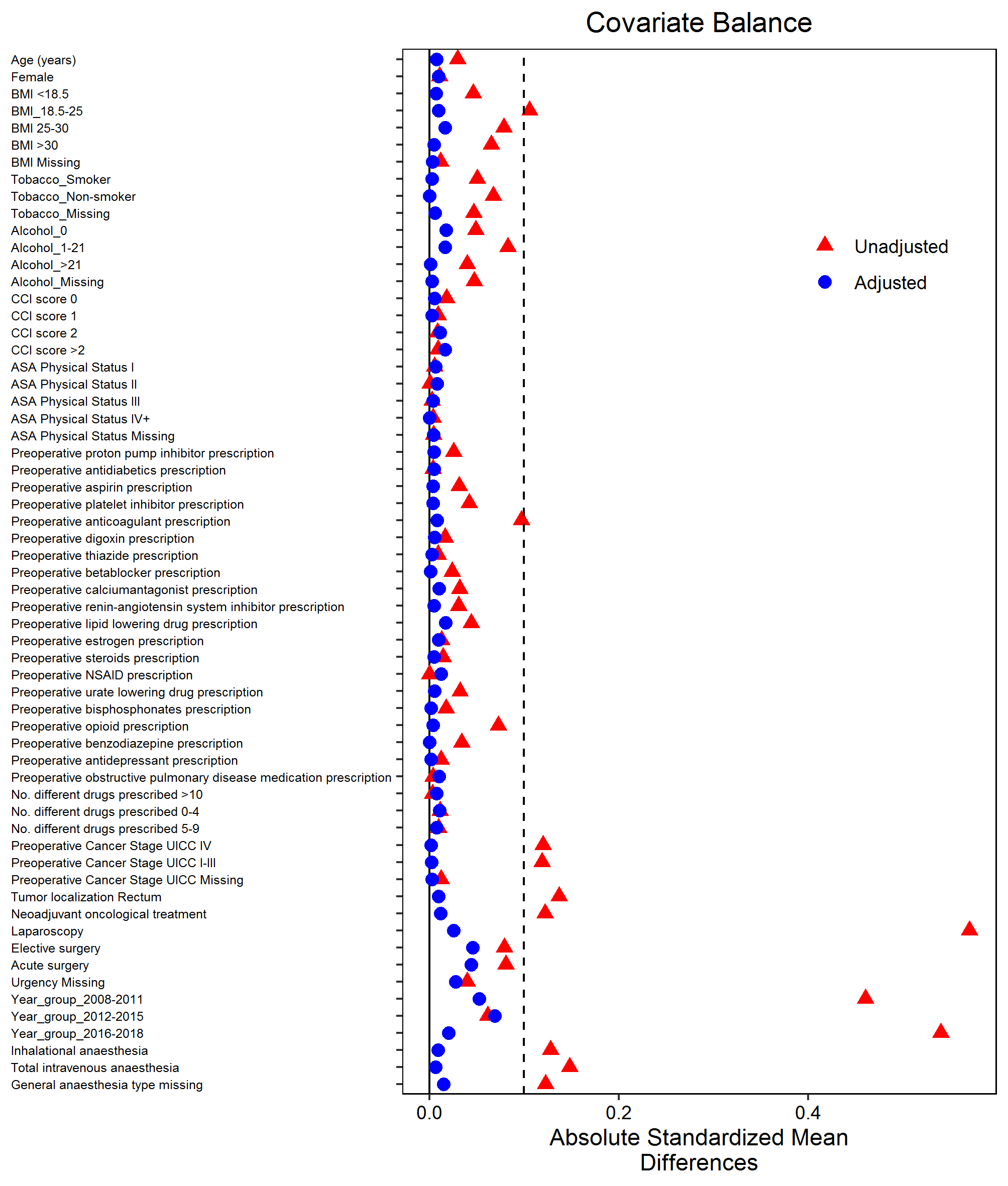

Supplement: Supplementary file 5 — Figure S5 [file AAS-66-869-s010.tif]

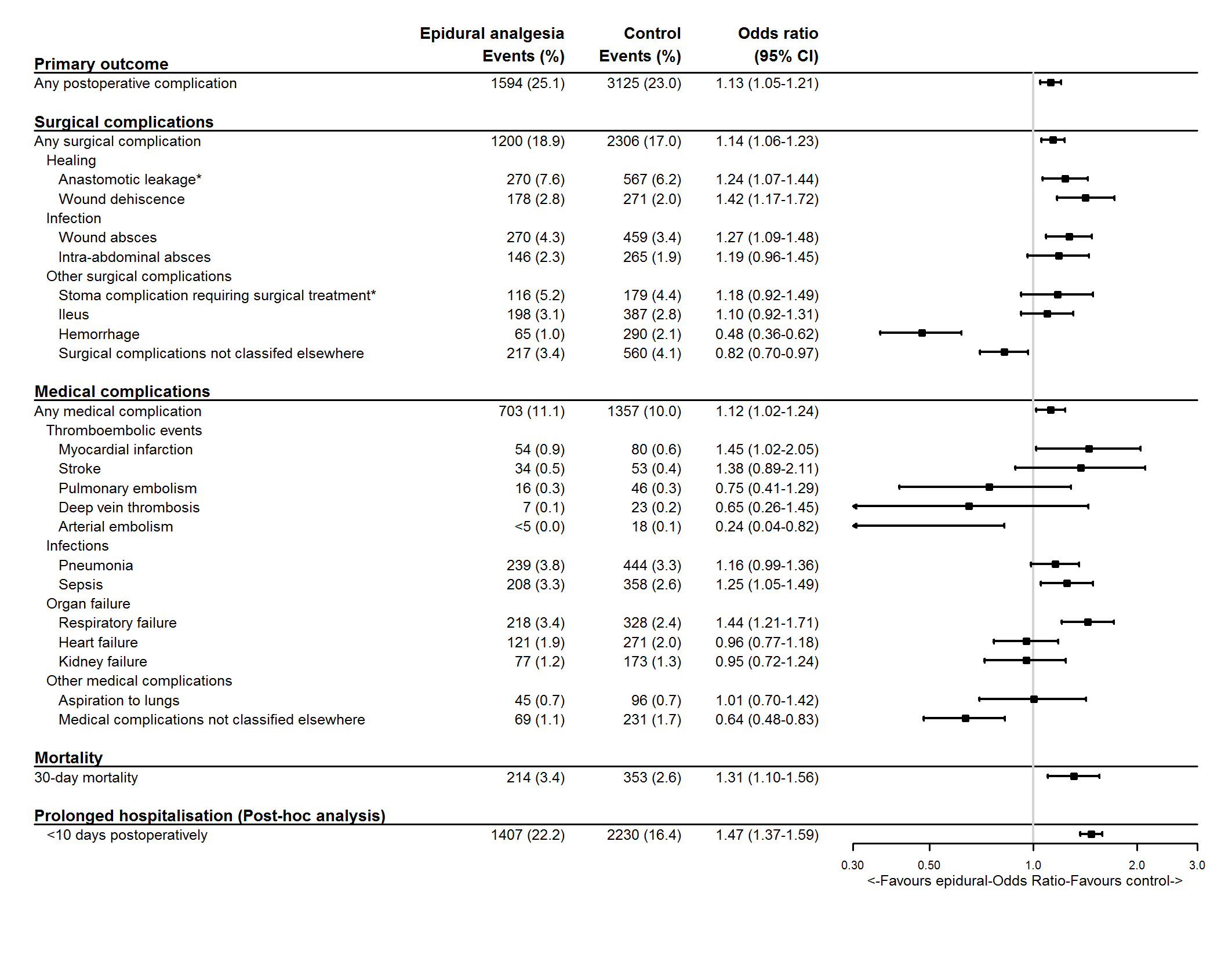

Supplement: Supplementary file 6 — Figure S6 [file AAS-66-869-s001.tif]

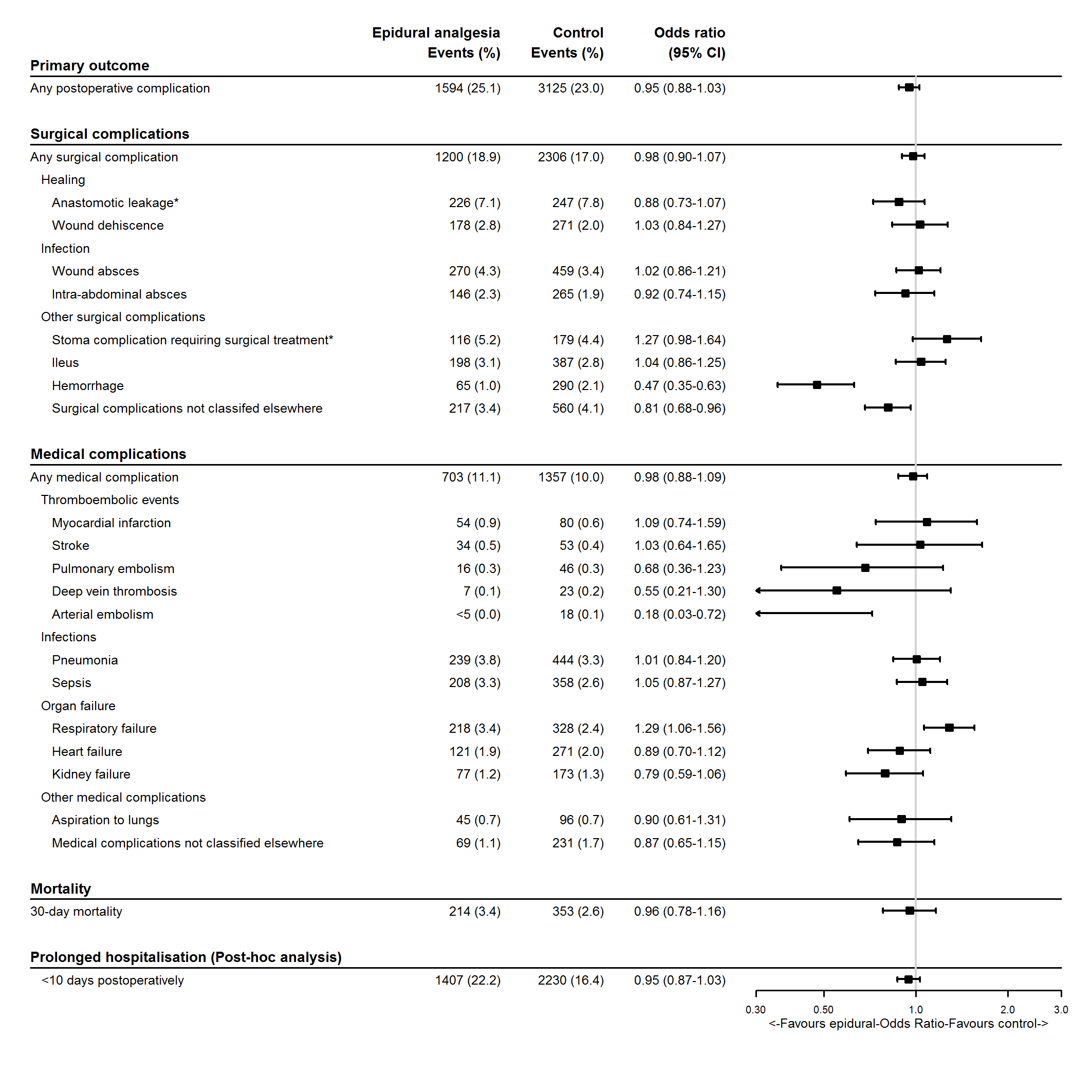

Supplement: Supplementary file 7 — Figure S7 [file AAS-66-869-s004.tif]

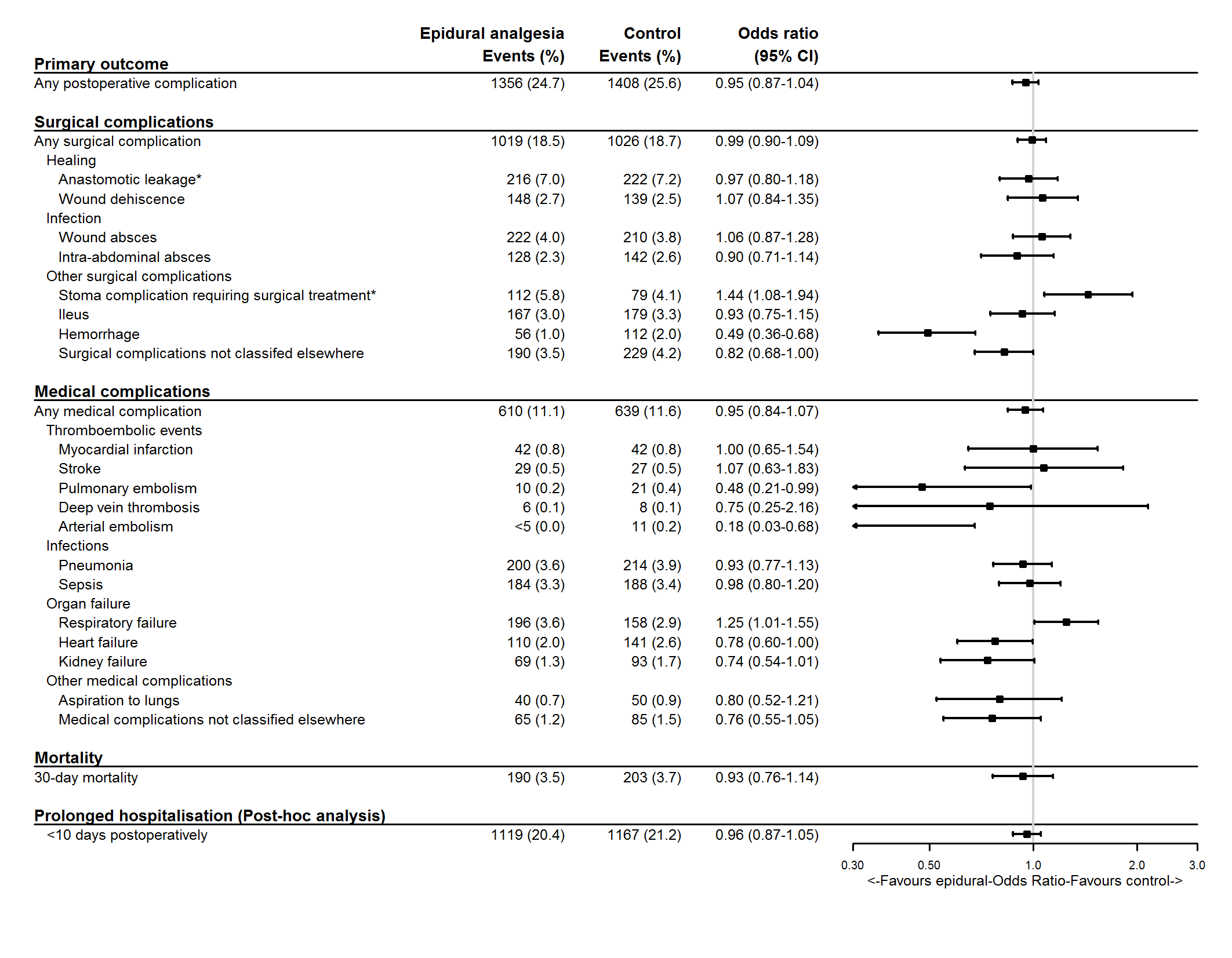

Supplement: Supplementary file 8 — Figure S8 [file AAS-66-869-s002.tif]

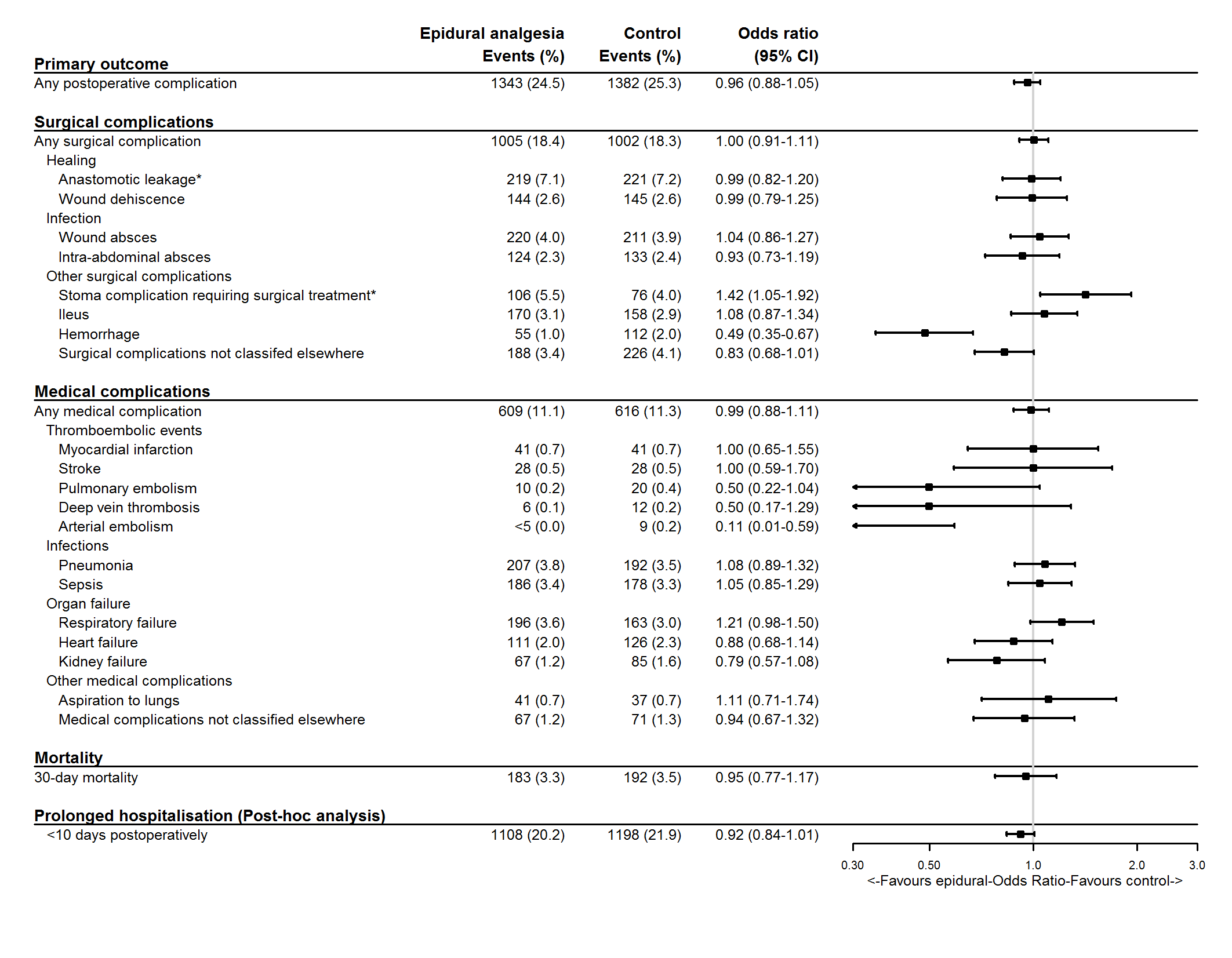

Supplement: Supplementary file 9 — Figure S9 [file AAS-66-869-s006.tif]
