# Supplementary material for: Epidural analgesia and postoperative complications in colorectal cancer surgery. An observational registry‐based study
Source: Acta Anaesthesiol Scand. 2022 Jun 27;66(7):869–79. doi: 10.1111/aas.14101 (PMC9543440; doi:10.1111/aas.14101)
Supplement: Supplementary file 11 — Table S2 [file AAS-66-869-s009.docx]

| **Table S2.** Covariate and outcome definitions | | |
| --- | --- | --- |
|  | Database | Definition |
| **Exposure** |  |  |
| Epidural analgesia | Danish Anaesthesia Database | Preoperative insertion of epidural catheter registered by anaesthetist. |
| **Baseline covariates** |  |  |
| Age | Danish Civil Registration System | Age in years at day of operation |
| Sex | Danish Civil Registration System | Male/female |
| Body Mass Index | Danish Anaesthesia Database | kg.m^-1^ (<18.5/18.5-25/25-30/>30) |
| Charlson Comorbidity Index | Danish Colorectal Cancer Group Database | Computation based on registrations in Danish National Patient Registry 10 years before diagnosis. (0/1-2/>2) |
| ASA Physical Status | Danish Anaesthesia Database | I/II/III/IV-V |
| Tobacco | Danish Colorectal Cancer Group Database | Registered by surgeon. Obtained Danish Anaesthesia Database if missing in Danish Colorectal Cancer Group Database (smoker/non-smoker) |
| Alcohol consumption | Danish Colorectal Cancer Group Database | Obtained from Danish Anaesthesia Database if missing in Danish Colorectal Cancer Group Database (0/1-21/>21 weekly units.) |
| Proton pump inhibitors | Danish National Prescription Registry | Filled prescription of ATC code “A02BC” within 3 month prior to surgery |
| Antidiabetics | Danish National Prescription Registry | Filled prescription of ATC code “A10” within 3 month prior to surgery |
| Acetyl Salicylic acid | Danish National Prescription Registry | Filled prescription of ATC code “B01AC06” within 3 month prior to surgery |
| Other platelet inhibitors | Danish National Prescription Registry | Filled prescription of ATC code “B01AC” (except “B01AC06”) within 3 month prior to surgery |
| Anticoagulants | Danish National Prescription Registry | Filled prescription of ATC code “B01A” (except “B01AC”) within 3 month prior to surgery |
| Digoxin | Danish National Prescription Registry | Filled prescription of ATC code “C01AA05” within 3 month prior to surgery |
| Thiazides | Danish National Prescription Registry | Filled prescription of ATC code “C03” within 3 month prior to surgery |
| Beta blockers | Danish National Prescription Registry | Filled prescription of ATC code “C07” within 3 month prior to surgery |
| Calcium channel blockers | Danish National Prescription Registry | Filled prescription of ATC code “C08” within 3 month prior to surgery |
| Drugs acting on renin angiotensin system | Danish National Prescription Registry | Filled prescription of ATC code “C09” within 3 month prior to surgery |
| Lipid lowering drugs | Danish National Prescription Registry | Filled prescription of ATC code “C10” within 3 month prior to surgery |
| Oestrogen hormone replacement | Danish National Prescription Registry | Filled prescription of ATC code “G03C” within 3 month prior to surgery |
| Corticosteroids for systemic use | Danish National Prescription Registry | Filled prescription of ATC code “H02” within 3 month prior to surgery |
| Non-steroid anti-inflammatory drugs | Danish National Prescription Registry | Filled prescription of ATC code “M01A” within 3 month prior to surgery |
| Urate lowering drugs | Danish National Prescription Registry | Filled prescription of ATC code “M04” within 3 month prior to surgery |
| Bisphosphonates | Danish National Prescription Registry | Filled prescription of ATC code “M05BA” or “M05BB” within 3 month prior to surgery |
| Opioids | Danish National Prescription Registry | Filled prescription of ATC code “N02A” within 3 month prior to surgery |
| Benzodiazepines | Danish National Prescription Registry | Filled prescription of ATC code “N05CD” or “N05CF” within 3 month prior to surgery |
| Antidepressants | Danish National Prescription Registry | Filled prescription of ATC code “N06A” within 3 month prior to surgery |
| Drugs for obstructive airway diseases | Danish National Prescription Registry | Filled prescription of ATC code “R03” within 3 month prior to surgery |
| Number of different drugs dispensed during last 3 months | Danish National Prescription Registry | Number of different drug prescriptions within 3 months prior to surgery (0-4, 5-9, >10) |
| Preoperative Cancer Stage | Danish Colorectal Cancer Group Database | Based on registrations of metastases present preoperatively (UICC stage I-III/UICC stage IV) |
| Localization | Danish Colorectal Cancer Group Database | Colon/Rectum |
| Preoperative oncologic treatment | Danish Colorectal Cancer Group Database | Yes/No |
| Intended surgical approach | Danish Colorectal Cancer Group Database | Intended surgical approach registered by surgeon (minimally invasive/laparotomy) |
| Urgency | Danish Colorectal Cancer Group Database | Elective/acute |
| General anaesthesia type | Danish Anaesthesia Database | Inhalational/total intravenous anaesthesia |
| Year group | Danish Colorectal Cancer Group Database | Year surgery (2004-2008/2009-2012/2013-2018)) |
| **Outcomes** |  |  |
| Overall postoperative complications (primary outcome) | Danish Colorectal Cancer Group Database | Any postoperative complication 30 days postoperatively registered by surgeon performing surgery(Yes/No) |
| Postoperative Surgical complications | Danish Colorectal Cancer Group Database | Any postoperative surgical complication within 30 days surgery postoperatively registered by surgeon performing postoperatively registered by surgeon performing surgery (yes, no) |
| *Haemorrhage* | Danish Colorectal Cancer Group Database | Haemorrhage 30 days postoperatively registered by surgeon performing surgery (Yes/No) |
| *Wound dehiscence* | Danish Colorectal Cancer Group Database | Wound dehiscence complication 30 days postoperatively registered by surgeon performing surgery (Yes/No) |
| *Ileus* | Danish Colorectal Cancer Group Database | Ileus 30 days postoperatively registered by surgeon performing surgery (Yes/No) |
| *Wound Abscess* | Danish Colorectal Cancer Group Database | Abscess of surgical wound 30 days postoperatively registered by surgeon performing surgery (Yes/No) |
| *Intraabdominal abscess* | Danish Colorectal Cancer Group Database | Intraabdominal abscess 30 days postoperatively registered by surgeon performing surgery (Yes/No) |
| *Stoma complication* | Danish Colorectal Cancer Group Database | Any complication requiring surgical intervention elated to stoma within 30 days from surgery for patients with stoma registered by surgeon performing surgery (Yes/No) |
| *Anastomotic leak* | Danish Colorectal Cancer Group Database | Postoperative anastomotic leak within 30 days from surgery for patients with anastomosis registered by surgeon performing surgery (Yes/No) |
| *Other surgical complication* | Danish Colorectal Cancer Group Database | Any other postoperative surgical complications 30 days postoperatively (Yes/No) |
| Postoperative Medical Complications | Danish Colorectal Cancer Group Database | Any postoperative medical complication within 30 days postoperatively registered by surgeon performing surgery (Yes/No) |
| *Stroke* | Danish Colorectal Cancer Group Database | Stroke within 30 days postoperatively registered by surgeon performing surgery (Yes/No) |
| *Acute myocardial infarction* | Danish Colorectal Cancer Group Database | Acute myocardial infarction within 30 days postoperatively registered by surgeon performing surgery (Yes/No) |
| *Aspiration* | Danish Colorectal Cancer Group Database | Aspiration to lungs within 30 surgery registered by surgeon performing surgery (Yes/No) |
| *Pneumonia* | Danish Colorectal Cancer Group Database | Pneumonia within 30 days postoperatively registered by surgeon performing surgery (Yes/No) |
| *Heart failure* | Danish Colorectal Cancer Group Database | Heart failure within 30 days postoperatively registered by surgeon performing surgery (Yes/No) |
| *Pulmonary embolism* | Danish Colorectal Cancer Group Database | Pulmonary embolism within 30 days postoperatively registered by surgeon performing surgery (Yes/No) |
| *Respiratory insufficiency* | Danish Colorectal Cancer Group Database | Respiratory insufficiency within 30 days postoperatively registered by surgeon performing surgery (Yes/No) |
| *Kidney Failure* | Danish Colorectal Cancer Group Database | Kidney failure within 30 days surgery registered by surgeon performing surgery (Yes/No) |
| *Sepsis* | Danish Colorectal Cancer Group Database | Sepsis within 30 days postoperatively registered by surgeon performing surgery (Yes/No) |
| *Deep vein thrombosis* | Danish Colorectal Cancer Group Database | Deep vein thrombosis within 30 days postoperatively registered by surgeon performing surgery (Yes/No) |
| *Arterial embolism* | Danish Colorectal Cancer Group Database | Arterial embolism within 30 days postoperatively registered by surgeon performing surgery (Yes/No) |
| *Other medical complication* | Danish Colorectal Cancer Group Database | Other medical complication within 30 days postoperatively registered by surgeon performing surgery (Yes/No) |
| 30-day mortality | Danish Civil Registration System | Death registered within 30 days postoperatively (Yes/No) |
| Length of hospitalisation (post-hoc analysis) | Danish National Patient Registry | Days from operation date to registration of discharge out of hospital. Data was available for all admission with a registration of cancer diagnosis. |
| Prolonged hospitalisation | Danish National Patient Registry | Length of hospitalisation exceeding 10 postoperative days |
| ATC = The Anatomical Therapeutic Chemical Classification System, UICC = Union for International Cancer Control | | |
